# Subregional initiatives for Chagas disease. A path of technical cooperation, opened by the countries, as an approach to a neglected disease

**DOI:** 10.1590/0074-02760210130chgsa

**Published:** 2022-07-06

**Authors:** José Fiusa Lima

**Affiliations:** 1 Associação de Antigos Funcionários das Nações Unidas

These considerations are made in the sense of contributing to suggest and / or clarify some aspects that are relevant to such an important public health issue in the Region of the Americas, which have been requested by the Fundação Oswaldo Cruz-FIOCRUZ Publication Secretariat. First, we must congratulate the authors for the power of synthesis, which allows us to have an overview of these regional initiatives in the article: “Surveillance and control of Chagas disease in the Americas: regional initiatives and the practical impossibility of interrupting the *Trypanosoma cruzi* transmission vector”.

Initially, it is necessary to clarify that the first Subregional initiative created by the Ministers of Health of the Cone Initiative Sur (INCOSUR) countries (Argentina, Bolivia, Brazil, Chile and Paraguay) in 1991 had the following objectives:

(i) Elimination of *Triatoma infestans* from homes and their peridomiciliary areas in endemic and probably endemic areas;

(ii) Reduction and elimination of domestic infestations of other species of triatomines that compete in the same areas occupied by *T. infestans*;

(iii) Reduction and elimination of transfusion transmission, by strengthening the effective selection of blood donors from the Network of Blood Banks.

Observations

(1) The title of this article literally says: “Regional initiatives and the practical impossibility of interrupting the transmission vector of *Trypanosama cruzi*”. It is necessary to clarify that the main objective of the Southern Cone Initiative was the elimination of *T. infestans*, the main vector in large areas of the Americas, especially in the INCOSUR. This initial objective, with the progress of work and the accumulation of experience, was difficult to achieve for all areas, but that did not stop progress, and under the “strategic construction” of the “interruption of vector transmission”, health was continued to be protected, lives and quality of life of millions of people, with effectiveness, efficiency and political initiative.

(2) When in 1991, the efforts of countries, experts, researchers, public health, research and assistance institutions began, with the Pan American Health Organization (PAHO) and the World Health Organization (WHO), to generate an Active and decisive technical cooperation among developing countries (as it was labeled at that time), the epidemiological reality and the conjunctural context that existed in the Region, around Chagas disease, was quite different.

The effort resulted in the creation of subregional spaces for technical cooperation between countries, South - South, with a triangular cooperation design when PAHO assumed the Technical Secretariat. The first effort implemented in 1992 was the INCOSUR/Chagas, built in the international health policy space of the Southern Cone Initiative in Health formed by Argentina, Bolivia, Brazil, Chile, Paraguay and Uruguay.

Its launch, success of activities and, fundamentally, its ability to convene in the countries a political will to assign marginal economic resources to control Chagas disease (until then orphaned of any assignment in many countries), promoted the creation of other subregional spaces to build through technical cooperation, prevention, control and care of American trypanosomiasis. This led to, in 1996 the Central American Initiative (IPCA) which in 2013 with the accession of Mexico became IPCAM, in 1997 the Andean Initiative, and in 2004 the Amazon Initiative (AMCHA).

(3) Each and every one of these Subregional Initiatives progressed and succeeded at their own pace, and with their own characteristics of implementation, materialisation of actions, development of evaluations, and national and international recognition.

It is fair to recognise that the action of each endemic country, all organised in their corresponding Subregional Initiative, often together with concomitant positive external factors of socio-economic-cultural transformation, obtained as demonstrable achievements today:

- overall decrease in the prevalence of human infection;

- overall decrease in the incidence of cases;

- decrease in morbidity and mortality from Chagas disease in many areas;

- new generations from many countries, free of infection or with a very low prevalence;

- great contributions to safe blood in blood banks throughout the Region, with universal donor screening for Chagas disease or its sustainability;

- support for the consideration and approach of the congenital Chagas problem within the enhanced transtheoretical model intervention (ETMI) plus strategy promoted by PAHO;

- visibility of the public health problem, to force its recognition at the political and decision-making level and effective instances of prevention, control and care;

- presentation and initiation of effective actions for sufficient, adequate and appropriate care for Chagas disease incorporated into the tasks of each National Health System;

- effective increase in the possibilities of financing actions for the prevention, control and care of Chagas by national funds and in the countries eligible to receive them, by international funds.

(4) But all this flow rate of protected lives and quality of life is not only a biological, eco-biological or biological construction, it is a comprehensive public health problem whose solutions go through political, economic, social, and cultural aspects of a neglected and silent disease, in addition to an invisible or overshadowed problem.

That was the great role of the Subregional Initiatives, and ultimately of the wisdom of the countries in using this international cooperation mechanism, to build in the union of their actions and decisions the effective areas of action that until the beginning of the nineties did not exist.

(5) Today’s Subregional Chagas Initiatives, as is and as they are, may not represent the ideal mechanism to continue advancing in prevention, control and care of Chagas. They have protected the countries’ activity on the subject during the current Coronavirus disease 2019 (COVID-19) pandemic, continuing their operation and guaranteeing actions that the countries have managed to maintain with great effort, some of them with notable progress.

(6) But if in the period 1991-2021, the Subregional Initiatives and all the mobilisation of resources and knowledge that they generated, made it possible to achieve the goals and objectives, totally or partially, that were achieved in all endemic countries, there is no reason to imagine that they will remain static, immutable and without any transformation by 2030. It is reasonable, logical and feasible to think that the same as active technical cooperation schemes between countries, if they continue to count on intergovernmental support and the contribution of the endemic countries of the Region, they will achieve the active profile and modality of action necessary to achieve the proposed goals.

If not, they will continue to be the best-known ideal mechanism for millions of people to be protected and cared for from Chagas disease.

Let us also count on the reinforcement of applied research, which serves to reinforce actions in its tools, methods or management, as well as with a renewed interdisciplinary participation of sectors and areas that were once distant from the subject: maternal and child health, internal medicine, pediatrics, transplant medicine, among many others.

The importance of the Subregional Chagas Initiatives should not be considered lightly or relativised, which is to consider the capacity for action and decision of the countries involved themselves to be surpassed, when they have been so important for the health of so many people, and until now there is no substitute idea or plan capable of protecting what has been achieved, projecting and executing what must be done today, and projecting what must be done and achieved in the future. Absolutely perfectible, but not expendable.
